# The effect of maternal intravenous hydration on amniotic fluid index in oligohydramnios

**DOI:** 10.1186/s13104-022-05985-6

**Published:** 2022-03-07

**Authors:** Fatemeh Azarkish, Roksana Janghorban, Shirin Bozorgzadeh, Abedeh Arzani, Rahemeh Balouchi, Mahnaz Didehvar

**Affiliations:** 1 Tropical and Communicable Diseases Research Center, Iranshahr University of Medical Sciences, Iranshahr, Iran; 2 Department of Midwifery, Iranshahr University of Medical Sciences, Iranshahr, Iran; 3 Department of Midwifery, School of Nursing and Midwifery, Maternal-Fetal Medicine Research Center, Shiraz University of Medical Sciences, Shiraz, Iran; 4 Department of Obstetrics and Gynecology, Iranshahr University of Medical Sciences, Iranshahr, Iran; 5 Department of Midwifery, Iranshahr University of Medical Sciences, Iranshahr, Iran; 6 Department of Health, Iranshahr University of Medical Sciences, Iranshahr, Iran

**Keywords:** Mothers, Intravenous hydration, Oligohydramnios

## Abstract

**Objective:**

Assessing amniotic fluid determines an important dimension of fetal health. Significant relationships of oligohydramnios, which occurs in 1–2% of pregnancies, with abnormal pregnancy outcomes such as intrauterine growth retardation (IUGR), fetal anomalies, umbilical cord compression, fetal distress, preterm childbirth, meconium-stained amniotic fluid, perinatal mortality and cesarean section necessitate the measurement of amniotic fluid volume in many prenatal fetal health assessments.

Amniotic fluid volume may significantly fluctuate between different gestational ages. Reduced uteroplacental perfusion can cause oligohydramnios by decreasing fetal renal blood flow and urine output. The appropriate management of isolated term oligohydramnios (ITO) is controversial. This study was performed aimed to assess the effect of maternal intravenous hydration on amniotic fluid index in oligohydramnios.

**Result:**

Forty-eight hours after completing fluid therapy, statistically significant differences were observed in the mean AFI in the intervention group (4.06 ± 0.33) and the control group (3.61 ± 0.35) (P < 0.0001) and also between the intervention group (0.532 ± 0.45) and the controls (−0.036 ± 0.18) (P < 0.0001).

**Conclusion:**

The results of the present study suggested that maternal intravenous hydration significantly increases AFI in women with oligohydramnios.

## Introduction

Assessing amniotic fluid determines an important dimension of fetal health. Significant relationships of oligohydramnios, which occurs in 1–2% of pregnancies [1], with abnormal pregnancy outcomes such as intrauterine growth retardation (IUGR), fetal anomalies, umbilical cord compression, fetal distress, preterm childbirth, meconium-stained amniotic fluid, perinatal mortality and cesarean section necessitate the measurement of amniotic fluid volume in many prenatal fetal health assessments [2]. Amniotic fluid volume may significantly fluctuate between different gestational ages. Reduced uteroplacental perfusion can cause oligohydramnios by decreasing fetal renal blood flow and urine output. The appropriate management of Isolated term oligohydramnios (ITO) is controversial [2]. According to Patrelli et al., maternal oral hydration significantly increases Amniotic fluid Index (AFI) in women with a normal AFI [3]. Umber also found fluid therapy not to be as effective as maternal oral hydration therapy in increasing AFI [4].

Despite the application of amnioinfusion in managing pregnant women with oligohydramnios, this method suffers certain limitations, including its exclusive application during labor, requiring fixed catheters and constant monitoring and its potential risks as a typical invasive procedure [5, 6].

Treatment of oligohydramnios includes hospitalization, specialist consultation, bed rest, oral rehydration and intravenous (I/v) hydration [7]. The effects of oral and infusion fluid therapies on AFI have been differently reported in literature [8]. There is a lack of trials which have reported maternal hydration with intravenous infusions (infusion of 3 liters of an isotonic Ringer’s lactate solution within 24 h) in oligohydramnios. To look upon this controversy, the aim of the present study to appreciate the effect of I/V hydration upon amniotic fluid volume [9]. In addition, maternal intravenous hydration in oligohydramnios has never been studied in Iranshahr, Iran, a tropical city with a hot and dry climate. The current clinical trial was conducted in 2016–2018 after receiving the approval of the Ethics Committee of the Iranshahr University of Medical Sciences with the aim to determine the effect of maternal intravenous hydration on amniotic fluid index in oligohydramnios.

## Main text

### Methods

This double-blind randomized clinical trial study was performed in IRAN Hospital of Iranshahr in 2016–2018. All the pregnant women diagnosed with oligohydramnios defined as AFI ≤ 5 cm who presented to IRAN Hospital in Iranshahr were investigated in terms of eligibility for the study.

The inclusion criteria were a singleton pregnancy, gestational age of at least 35 weeks (based on the last menstrual period and confirmed by the results of ultrasound or determined through early pregnancy sonography), healthy fetal membranes and no evidence of fetal distress in nonstress test, structural abnormalities and fetal distress. Mothers with confounding factors such as post-term pregnancy, hyperthyroidism, diabetes, hypertension, heart disease, liver disease, kidney disease and amniorrhexis were excluded from the study.

The eligible mothers were included in the study after explaining them about the study objectives, methods, its benefits and potential disadvantages; all the participants signed the written consent forms. The sample size was calculated as 30 per group based on a pilot study. The subjects were randomly assigned to the experimental group (infusion of 3 liters of an isotonic Ringer’s lactate solution within 24 hours) and the control group (constant-rate fluid infusion to keep vein open). AFI was measured through ultrasound before and 24 after the end of maternal intravenous hydration by using a 3.5 MHz transducer by a specialist.Re-assessment was 24 h after the end of maternal intravenous hydration.

The patients had empty bladders at the time of remeasurement of amniotic fluid index.

The control group received 2000 ml of Ringer’s serum as keep vein open (KVO) at first use within 24 h. In the intervention and control groups, the rates of oral absorption of fluids during the intervention were not meaningful.

All the mothers were hospitalized and their vital signs were regularly monitored. A sonographer performed ultrasound before and after fluid therapy in both groups.

The sonographer and the data analyst were blinded to the grouping of the mothers.

To avoid bias, the ultrasound of each mother before and after the study was performed by the same sonographer. Randomization was performed by someone outside the treatment team. However, it was not possible for mother and the therapist to blind.

**Fig. 1:**
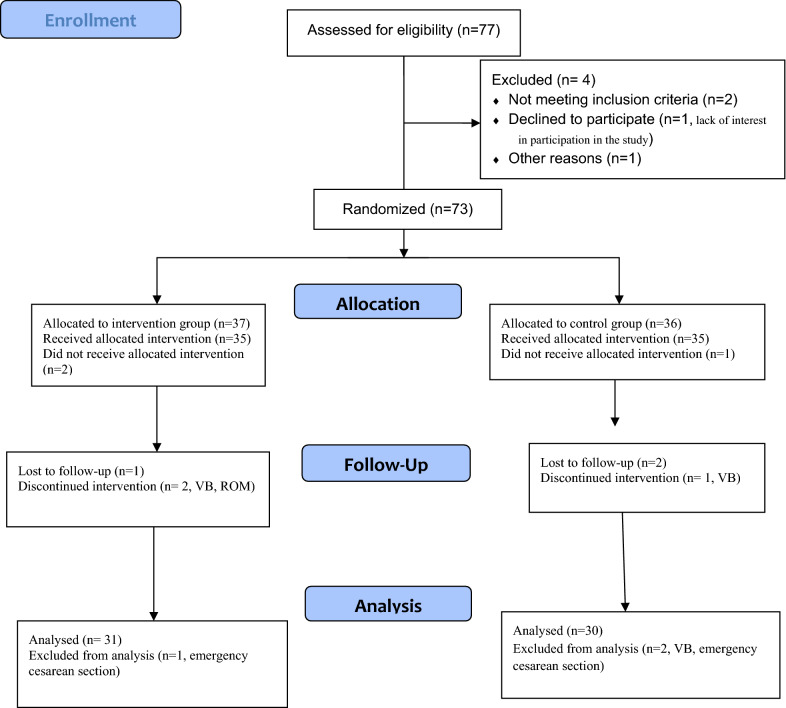
CONSORT flow diagram of patients through the study

Estimation of AFI before and after the intervention was performed in both groups by a specialist. After completing the study, pregnancy termination was considered for the mothers based on their gestational age and AFI (Fig. 1).

Data were collected using a questionnaire containing the demographic details and ultrasound results of the mothers before and after fluid therapy were compared within the groups using the paired t-test and between the groups using the t-test. P < 0.05 was set as the level of statistical significance. The present study was approved by the Ethics Committee (IR.IRSHUMS.REC.1394.11) and registered at Registry of Clinical Trials (code: IRCT20160418027464N3).

### Results

Among 77 mothers with oligohydramnios, 61 mothers were eligible to the include in the study and were randomly assigned to the intervention group (n = 31) and the control group (n = 30). The two groups were matched in terms of age, parity, gestational age and birth weight of neonates. No statistically significant differences were observed between the two groups in terms of frequency of cesarean section (Table 1).

**Table 1:** Baseline characteristics of hydrotherapy and control groups

Characteristics	Hydrotherapy group (mean ± SD)	Control group (mean ± SD)	P-value
Maternal age (Years, mean ± SD)	22.56 ± 3.77	21.52 ± 4.62	0.388
Parity (NO)	1.56 ± 0.65	1.64 ± 0.75	0.690
Gestational age (WK, mean ± SD)	39.44 ± 1.12	39.60 ± 0.81	0.567
Birth wight (Grams, mean ± SD)	3014 ± 306.36	3026 ± 199.01	0.870
Cesarean section (NO, %)	1 (33.3%)	2 (66/7%)	0.552

The difference between weight and BMI in the two groups was clinically inconsiderable.Before maternal intravenous hydration, insignificant differences were observed between the intervention and control groups in terms of the mean AFI (P =  0.128). Forty-eight hours after completing fluid therapy, statistically significant differences were observed in the mean AFI between the two group (P < 0.0001) (Table 2).

**Table 2:** AFI changes within and between the hydrotherapy and control groups

Characteristics	Hydrotherapy group (n = 25)	Control group (n = 25)	P-value	Cohen's d estimate (95% Conf. Interval)
AFI before intervention (Cm,mean ± SD)	3.49 ± 0.34	3.64 ± 0.36	0.128	–
AFI after intervention (Cm,mean ± SD)	4.06 ± 0. 33	3.61 ± 0. 35	0.000	1.499 (0.92; 2.06)

No changes in blood pressure were reported during hydration. No participants were excluded from the study owing to the severe effects of the intervention.

## Discussion

According to the results of the present maternal hydration significantly increased AFI in women with oligohydramnios. Kiran et al. found acute maternal hydration to increase amniotic fluid volume [7]. In line with the present study, numerous studies have demonstrated the effectiveness of maternal hydration in treating oligohydramnios [8–10].

Lorzadeh et al. found that maternal intravenous fluid therapy more significantly increased AFI; when compared to oral fluid therapy, which is consistent with the present findings [11]. Hofmeyr found that simple maternal fluid therapy is effective in improving oligohydramnios by increasing amniotic fluid volume, especially before performing an ECV, which is consistent with the findings of the present study [12]. Magann et al. determined the amniotic fluid volume by dye dilution technique before cesarean delivery, IV fluids were given and the amniotic fluid volume was measured at the cesarean delivery. The comparison between the volumes before and after cesarean to the volume after cesarean showed that IV fluids increased the actual volume [13] .

Malhotra et al. reported an increase in AFI one hour after acute fluid therapy and no significant increases 24 and 48 h after the intervention compared to the initial hours of the treatment [14]. The mechanism of altering AFI after maternal hydration and the duration of its increase remain unclear. Fetal urine production plays a key role in amniotic fluid volume after 24 weeks of gestation. Increasing osmolality and intravascular volume can increase fetal diuresis. Different clinical trials suggested the responsiveness of the fetus to changes in maternal intravascular volume and osmolality [15]. Kim et al. found maternal oral fluid therapy to facilitate the transfer of water to the fetus and increase fetal urine production [5]. Ali et al. reported a significant increase in AFI after the intravenous infusion of one liter of fluid in amniorrhexis, although they failed to clarify the etiology of the hydration-associated increase [16].

Flack et al. reported insignificant changes in fetal urine production after maternal oral fluid therapy in women with oligohydramnios [17]. Soni et al. found the intravenous administration of arginine vasopressin as an agonist in mothers to increase fetal urine production, reduce fetal swallowing activity and ultimately significantly increase amniotic fluid volume [18]. According to Yan Rosenberg et al., hydration alone cannot increase AFI in oligohydramnios in term gestational ages. They attributed the changes in AFI to other factors such as daily physiological variations in AFI, changes in fetal presentation and inaccurately measuring of AFI [19]. In the present study population comprised of women with gestational age of at least 35 weeks who had idiopathic oligohydramnios. Women whose oligohydramnios was associated with IUGR, amniorrhexis and fetal anomalies were excluded. The present study re-measured AFI 24 h after fluid therapy. Labor should be performed in subjects with a persistently-low AFI. Maternal intravenous hydration is recommended to be administered in oligohydramnios cases given the associated increase in AFI as observed in the present research. Maternal hydration therefore definitely plays a key role in improving AFI in patients with oligohydramnios.

Given the complications and numerous maternal and fetal problems caused by oligohydramnios and the increase in AFI caused by maternal intravenous hydration, with no maternal complications, this treatment is recommended for mothers with Isolated term oligohydramnios (ITO). More studies are needed to perform in order to achieve clinical results

## Conclusion

According to the findings of study, Maternal Intravenous Hydration significantly increased amniotic fluid index in in women with oligohydramnios. Researchers are recommended to do further studies with a larger sample size and Meta-Analysis in the future

## Limitation

One of the main limitations of the present study was the small sample size which makes it hard to generalize the obtained results.

## Data Availability

The study protocol and data collection forms are available from the corresponding author (RJ) upon reasonable request.

## Abbreviations

IUGR: Intrauterine growth retardation
ITO: Isolated term oligohydramnios
AFI: Amniotic fluid index
I/V: Intravenous
ECV: External cephalic version
